# Removal of endothelial surface-associated von villebrand factor suppresses accelerate datherosclerosis after myocardial infarction

**DOI:** 10.1186/s12967-024-05231-6

**Published:** 2024-05-01

**Authors:** Koya Ozawa, William Packwood, Matthew A Muller, Yue Qi, Aris Xie, Oleg Varlamov, Owen J. McCarty, Dominic Chung, José A. López, Jonathan R. Lindner

**Affiliations:** 1 Sydney Medical School Nepean, Faculty of Medicine and Health, Department of Cardiology, The University of Sydney, Nepean Hospital, Sydney, NSW Australia; 2 Knight Cardiovascular Institute, Oregon Health & Science University, Portland, OR USA; 3 Cardiovascular Division and Robert M. Berne Cardiovascular Research Center, University of Virginia, Box 801394, 415 Lane Rd, Charlottesville, VA 22908 USA; 4 Oregon National Primate Research Center, Portland, OR USA; 5 Department of Biomedical Engineering, Oregon Health & Science University, Portland, USA; 6 BloodWorks Research Institute, University of Washington, Seattle, WA USA

**Keywords:** Atherosclerosis, Molecular imaging, Platelets, Von Willebrand factor

## Abstract

**Background:**

Thromboinflammation involving platelet adhesion to endothelial surface-associated von Willebrand factor (VWF) has been implicated in the accelerated progression of non-culprit plaques after MI. The aim of this study was to use arterial endothelial molecular imaging to mechanistically evaluate endothelial-associated VWF as a therapeutic target for reducing remote plaque activation after myocardial infarction (MI).

**Methods:**

Hyperlipidemic mice deficient for the low-density lipoprotein receptor and Apobec-1 underwent closed-chest MI and were treated chronically with either: (i) recombinant ADAMTS13 which is responsible for proteolytic removal of VWF from the endothelial surface, (ii) N-acetylcysteine (NAC) which removes VWF by disulfide bond reduction, (iii) function-blocking anti-factor XI (FXI) antibody, or (iv) no therapy. Non-ischemic controls were also studied. At day 3 and 21, ultrasound molecular imaging was performed with probes targeted to endothelial-associated VWF A1-domain, platelet GPIbα, P-selectin and vascular cell adhesion molecule-1 (VCAM-1) at lesion-prone sites of the aorta. Histology was performed at day 21.

**Results:**

Aortic signal for P-selectin, VCAM-1, VWF, and platelet-GPIbα were all increased several-fold (*p* < 0.01) in post-MI mice versus sham-treated animals at day 3 and 21. Treatment with NAC and ADAMTS13 significantly attenuated the post-MI increase for all four molecular targets by > 50% (*p* < 0.05 vs. non-treated at day 3 and 21). On aortic root histology, mice undergoing MI versus controls had 2–4 fold greater plaque size and macrophage content (*p* < 0.05), approximately 20-fold greater platelet adhesion (*p* < 0.05), and increased staining for markers of platelet transforming growth factor-β1 signaling. Accelerated plaque growth and inflammatory activation was almost entirely prevented by ADAMTS13 and NAC. Inhibition of FXI had no significant effect on molecular imaging signal or plaque morphology.

**Conclusions:**

Plaque inflammatory activation in remote arteries after MI is strongly influenced by VWF-mediated platelet adhesion to the endothelium. These findings support investigation into new secondary preventive therapies for reducing non-culprit artery events after MI.

**Supplementary Information:**

The online version contains supplementary material available at 10.1186/s12967-024-05231-6.

## Background

Arterial plaque development in atherosclerosis is caused primarily from vascular accumulation of certain modified lipoproteins (e.g. oxidized or dialylated low-density lipoprotein [LDL]) which triggers an innate immune system response with accumulation of monocyte-derived cells [1, 2]. Other contributing pathways for atherogenesis include thromboinflammation where immune cell recruitment is supported by platelet-endothelial interactions, or abnormalities in the adaptive immune response [3, 4]. Most of these processes are thought to be indolent. However, there is evidence that ischemia-reperfusion injury that occurs with myocardial infarction (MI) may accelerate these processes. Early after MI, risk for recurrent events in non-culprit territories is heightened despite the advent of secondary preventive therapies [5]. The production, reprogramming, and release of innate immune cells after MI contributes to this risk [6]. MI also triggers the release of alarmins and an increase in circulating biomolecules of the damage-associated molecular pattern (DAMP) family [7, 8]. These factors are likely involved in the post-MI accelerated growth and adverse phenotypic change of non-culprit plaques that have been observed by histology in animal models [6, 9], and by serial intravascular ultrasound in patients after ST-elevation MI [10].

In vivo molecular imaging has previously provided temporal information on the global pro-inflammatory endothelial responses that occur after a focal ischemic event. On molecular imaging of remote arteries after MI, there is an early and sustained expression of the endothelial cell adhesion molecules P-selectin and vascular cell adhesion molecule-1 (VCAM-1) [9]. Sustained increases in endothelial surface-associated von Willebrand factor (VWF) and secondary platelet adhesion beyond that which occurs during indolent atherosclerosis has also been detected on arterial molecular imaging [9, 11, 12]. Oxidative stress is thought to be an important contributor to excess platelet adhesion post-MI based on its ability to impair the action of ADAMTS13 (A Disintegrin-like and Metalloprotease domain with Thrombospondin type I motifs) to remove VWF from the endothelial surface after VWF is secreted from endothelial storage granules [9, 13]. Because platelets are a rich source of pro-inflammatory cytokines and growth factors [14–16], it is likely that thromboinflammation involving VWF-mediated platelet adhesion plays a major role in post-MI remote plaque activation.

In this study we applied molecular imaging to assess remote plaque activation, and to mechanistically investigate whether it can be inhibited by therapies that remove VWF from the endothelial surface, either proteolytically or through disulfide-bond reduction. In vivo contrast-enhanced ultrasound (CEUS) molecular imaging of endothelial phenotype and histology of plaque size, content, platelet signaling were used in atherosclerotic mice after MI to assess benefits from VWF-targeted interventions which included: (i) chronic administration of recombinant ADAMTS13 which proteolytically removes VWF from the endothelium, (ii) N-acetylcysteine (NAC) which removes VWF by disulfide bond reduction [17], , and (iii) inhibition of the coagulation pathway serine protease Factor XI (FXI) which has been shown to cleave ADAMTS13 at the C-terminal domain [18]. 

## Methods

### Animal models

The study was approved by the institutional Animal Care and Use Committee and conformed to guidelines in the PHS Guide for the Care and Use of Laboratory Animals. Mice with homozygous genetic deletion of both the low-density lipoprotein receptor and the Apobec-1 mRNA editing peptide for Apolipoprotein-B (*LDLR*^*-/-*^*Apobec1*^*-/-*^) were studied and were fed a standard laboratory diet (PicoLab 5L0D, LabDiet, Northlake, TX). These mice develop reproducible and indolent age-dependent atherosclerotic plaque at lesion-prone sites of the aorta on standard diets [19], making them well-suited for studying sudden interventions such as MI. Moreover, the deletion of Apobec-1 results in greater similarity to humans with respect to high circulating levels of ApoB100-containing lipoproteins [20]. Mice entered into the study at 20–25 weeks of age and mice of either sex were used based on the sex independent nature of disease development in this strain [20]. For all procedures, mice were anesthetized with inhaled isoflurane (1.0–2.0%) and kept euthermic. A jugular vein was cannulated for intravenous access when required for CEUS molecular imaging.

### Myocardial infarction

A closed-chest model of MI was used in order to minimize the confounding effects of thoracotomy-related inflammation on early post-MI molecular imaging studies [9]. At five to seven days prior to MI, mice were anesthetized, intubated, and placed on positive pressure mechanical ventilation. A left lateral thoracotomy was performed through which an 8 − 0 nylon suture was passed under the left anterior descending (LAD) coronary artery but was not tied. The free ends of the suture were exteriorized through the chest wall and left subcutaneously on layered closure. After 5–7 days, mice were anesthetized, and tension was placed on the exteriorized sutures for forty minutes, during which ST-segment elevation on electrocardiographic monitoring and wall motion abnormalities on high-frequency transthoracic two-dimensional echocardiography (Vevo 2100, Visualsonic Inc., Toronto, Canada) were used to confirm ischemia in the LAD territory. Non-ischemic sham-treated control animals had sutures placed without tightening (*n* = 25) and were studied at the same time intervals as animals undergoing MI. Naïve animals without surgical intervention were also studied for the molecular imaging component of the study (*n* = 14).

### Post-MI interventions

Mice were randomized to receive either no therapy or one of three therapies intended to reduce thromboinflammation. Recombinant human ADAMTS13 (rADAMTS13) was generated from Tet-On HEK293 cells transfected with the pNBioSec vector [21], and administered at a continuous rate of 2 µg/day for three weeks by osmotic minipump (model 1004, Alzet, Cupertino, CA) placed at the time of MI (*n* = 19). NAC (Sigma Aldrich, St. Louis, MO) was given orally for three weeks (600 mg/kg/day) by supplementing the drinking water at a concentration calibrated to intake (*n* = 19). FXI was inhibited by intraperitoneal administration of a monoclonal antibody (clone 14E11) (1 mg/kg) immediately after MI and on post-MI days 3, 9, and 15 (*n* = 4) [22]. Because of the differences in administration routes and vehicles for these therapies, non-treated mice undergoing MI did not receive any sham therapies.

### Aortic molecular imaging

CEUS molecular imaging of the proximal thoracic aorta, a remote atheroprone site, was performed at either day 3 or 21 after MI or sham procedure using microbubble probes targeted to either the A1-domain of VWF, GPIbα as an indicator of platelet adhesion, or to the extracellular domains of P-selectin or vascular cell adhesion molecule (VCAM)-1. These probes were constructed from biotinylated lipid-shelled decafluorobutane microbubbles that were prepared by sonication of gas-saturated aqueous lipid suspension of distearoylphosphatidylcholine (2 mg/mL), polyoxyethylene-40-stearate (1 mg/mL), and distearoylphosphatidylethanolamine-PEG (2000) biotin (0.1 mg/mL; Avanti Polar Lipids, Alabaster, AL). Biotinylated ligands were conjugated to the surface as described previously using a streptavidin bridge [19]. Ligands used for targeting were: dimeric recombinant murine VWF A1 domain (mature VWF amino acids 445 to 716) for targeting platelet GPIbα [9, 23]; a cell-derived biotinylated polypeptide representing the N-terminal 300 amino acids of GPIbα for targeting endothelial VWF; [23] and monoclonal antibodies against the extracellular domain of either P-selectin (RB40.34, BD Biosciences, San Jose, California), or VCAM-1 (clone 429, BD Biosciences). These targeted agents have been extensively validated in previous experiments using flow chamber adhesion to intended molecular targets, intravital microscopy observation of targeted attachment, ex vivo observation of aortic plaque attachment in shear flow, and targeted imaging in the absence and presence of specific inhibitors, cleavage enzymes (for VWF), and gene-targeted knockout models [12, 19, 23–26]. Control microbubbles were prepared with isotype control antibody (R3-34, BD Biosciences). Microbubble concentrations and size distributions were measured by electrozone sensing (Multisizer III, Beckman Coulter). CEUS was performed with a linear-array transducer and power-modulation pulse-inversion imaging (Sequoia, Siemens Medical Systems, Mountainview, California). Imaging was performed at 7 MHz, a dynamic range of 55 dB, and a mechanical index of 1.0. Gain was set at a level that eliminated pre-contrast background speckle and was kept constant. The ascending aorta was imaged in long-axis gated to end-diastole. Images were acquired 8 min after intravenous injection of 1 × 10^6^ targeted or control microbubbles performed in random order. Animals were randomized to receive three of the five microbubble agents according to safety-based limits on injected volumes. Signal intensity was measured using established protocols that differentiate signal from adherent versus circulating microbubbles [9, 19]. Regions-of-interest were standardized encompassing the aorta from the sinuses to the proximal arch.

### Echocardiography

High-frequency (40 MHz) transthoracic echocardiography (Vevo 2100, Visualsonics, Toronto, Canada) was performed in all control mice and all post-MI mice within 10 min of reperfusion, and at post-MI days 3 and 21. Parasternal long-axis and mid-ventricular short-axis views were obtained to measure left ventricular (LV) dimensions and volumes at end-systole and end-diastole, LV ejection fraction (LVEF), and limited-view wall motion score index. Stroke volume was calculated by the product of the LV outflow tract cross-sectional area and time-velocity integral on angle-corrected pulsed-wave Doppler. Cardiac output was calculated as the product of stroke volume and heart rate. Global longitudinal strain (GLS) and circumferential strain (GCS) were calculated using speckle-tracking echocardiography from mid-ventricular parasternal short-axis view and a modified apical view, and were quantified as the average of a six-segment model.

### Histology

Histology of the aortic root and the mid-ascending aorta for *LDLR*^*-/-*^*Apobec1*^*-/-*^ mice was performed 21 days after MI or sham procedure. Blood was removed from the arterial system by infusion of isothermic buffered saline after which the aorta was perfusion-fixed. Trans-axial sections of the aortic root at the level of the sinuses were stained with Masson’s trichrome to assess the plaque area within the internal elastic lamina, and collagen content. Immunofluorescence histology was performed with anti-mouse primary mAb against Mac-2 (M3/38, Invitrogen, Waltham, Massachusetts, USA) for monocytes/macrophages, against CD41 (ab181582, Abcam, Cambridge, United Kingdom) for platelets, and against matrix metalloproteinase-9 (MMP-9) (PA5-13199, ThermoFisher). Staining for evidence of vascular cell signaling through platelet-derived TGFβ1 was performed with mAb against β-catenin (51067-2-AP, Proteintech, Rosemount, IL) and phosphorylated SMAD2 (pSMAD2) (H.205.4, Invitrogen). Secondary staining was performed with species-appropriate secondary polyclonal antibodies labeled with ALEXA fluorochromes (Fluor-488, Fluor-568, or Fluor-647) and observations were made by confocal fluorescent microscopy (TCS SP5, Leica Microsystems, Buffalo Grove, IL). Spatial extent of plaque size, collagen content, or fluorescent staining area were quantified using Image-J.

### Arterial NF-κB

For assessment of treatment-related changes in arterial NF-κB, the proximal descending thoracic aorta was obtained immediately after euthanasia, homogenized in cell extraction solution, and incubated at 4° C for 20 min. After centrifugation at 4 °C for 20 min, the supernatant was stored at − 80° C. Murine NF-κB transcription factor complex p65 subunit was quantified by enzyme-linked immunosorbent assay (SimpleStep NF-κB-p65, pS536, Abcam). Data were normalized to tissue weight.

### Statistical analysis

Data were analyzed statistically using Prism version 9.0 (GraphPad La Jolla, CA). Continuous variables that were normally distributed are displayed as mean ± SD unless stated. Tests for normal versus non-normal distribution were made using a Shapiro-Wilk test. A Student *t* test was performed for comparisons of normally distributed data. For non-normally distributed data, Mann-Whitney *U* test was used as appropriate according to experimental conditions and data were displayed using box-whisker plots depicting median (bar), interquartile range (box), and range (whiskers). For multiple comparisons, one-way analysis of variance was performed for normally distributed data and a Kruskal-Wallis test was performed for non-normally distributed data. Post-hoc individual comparisons were performed using Dunn’s test for multiple comparisons. Differences were considered significant at *P* < 0.05.

## Results

### Acute myocardial ischemia

**Fig. 1:**
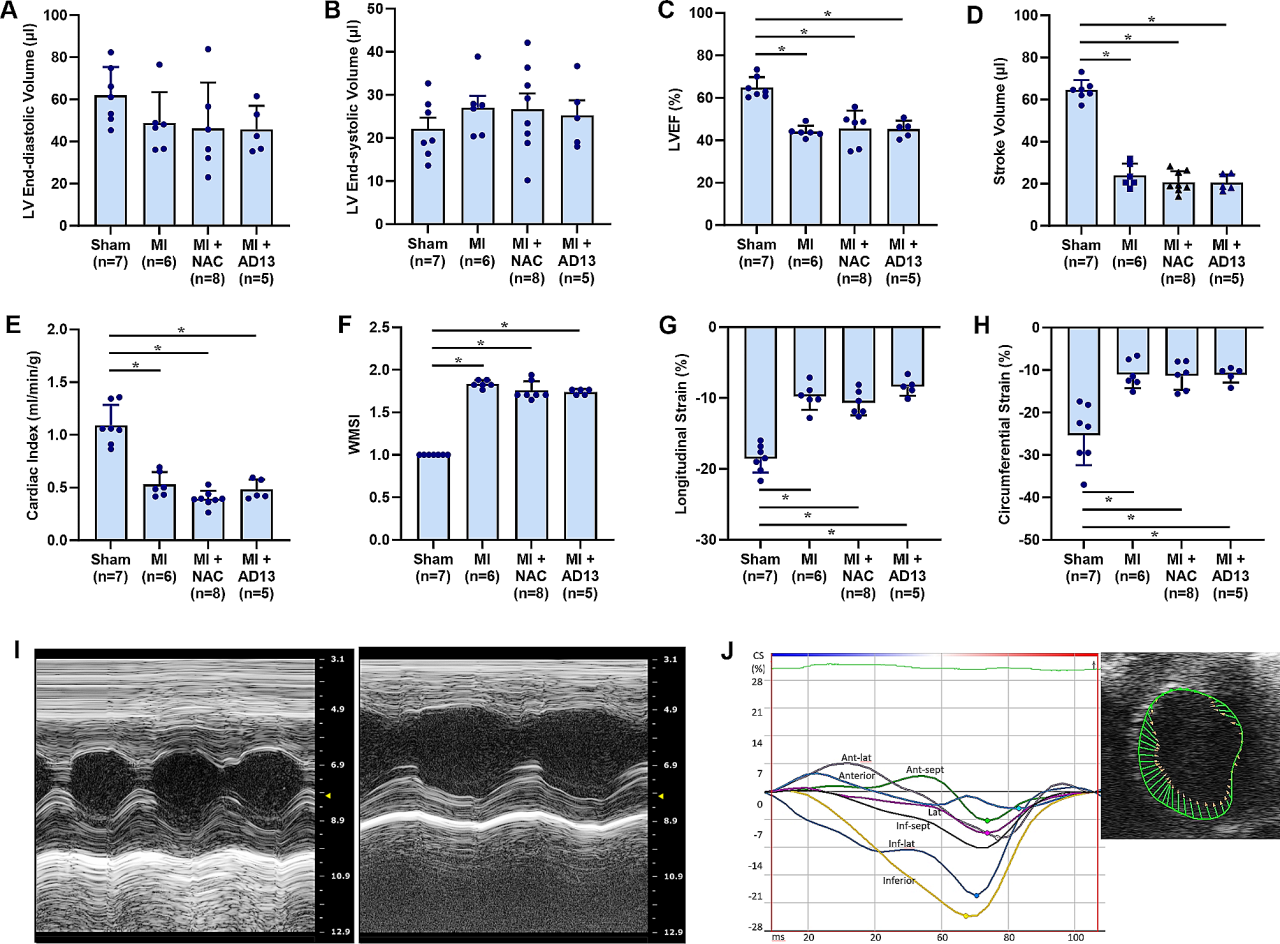
Left ventricular dimension and function by echocardiography after ischemia-reperfusion injury and in sham-treated dko mice. Mean (± SEM) values are shown for: (**A**) left ventricular (LV) end-diastolic volume, (**B**) LV end-systolic volume, (**C**) LV ejection fraction (LVEF), (**D**) stroke volume, (**E**) cardiac index, (**F**) wall motion score index (WMSI), (**G**) single-plane averaged longitudinal strain, and (**H**) single-plane averaged circumferential strain. Data shown are for mice undergoing sham suture placement, closed-chest MI, and closed-chest MI with treated with NAC or ADAMTS13. (**I**) Example of m-mode echocardiography taken from the mid-LV cavity parasternal short-axis plane from a DKO mouse (untreated) at baseline (left) and after MI (right). (**J**) Example of circumferential strain illustrating abnormal strain in the anterior-lateral segments on parasternal short-axis imaging. *p < 0.05 (by ANOVA corrected for multiple comparisons)

Myocardial ischemia was confirmed in all *LDLR*^*-/-*^*Apobec1*^*-/-*^ mice undergoing closed-chest LAD occlusion by the presence of both acute ST-segment elevations on electrocardiography and regional wall motion abnormalities on echocardiography (Supplemental Videos 1 to 4). These findings were not observed in any of the sham-treated animals. The severity and spatial extent of myocardial injury were evaluated by quantitative echocardiography performed immediately upon reperfusion. LV systolic function by LVEF, stroke volume index, cardiac index, wall motion score index, and single-plane averaged longitudinal and circumferential strain were abnormal in mice undergoing MI compared to sham-treated controls and were similar between all post-MI groups irrespective of therapy assignment (Fig. 1). These findings indicate that the degree of myocardial ischemic injury used to stimulate remote plaque activation was similar between the different groups undergoing MI. Animals treated with anti-FXI, which had a similar degree of injury, were not shown due to lack of beneficial response (below).

### Molecular imaging of post-MI thromboinflammation and VWF-targeted interventions

Aortic endothelial molecular imaging with CEUS and probes targeted to endothelial surface-associated VWF (A1 domain), platelet GPIbα, P-selectin, and VCAM-1 revealed a higher signal from these probes compared to control non-targeted probe in both naïve and sham-treated mice (Supplemental Fig. 1). These findings indicate that *LDLR*^*-/-*^*Apobec1*^*-/-*^ mice have an activated endothelial phenotype manifest by surface adhesion molecule expression, VWF mobilization, and platelet adhesion; and also demonstrate no further aortic endothelial activation from surgical placement of an LAD ligature 5–7 days earlier in the sham group.

**Fig. 2:**
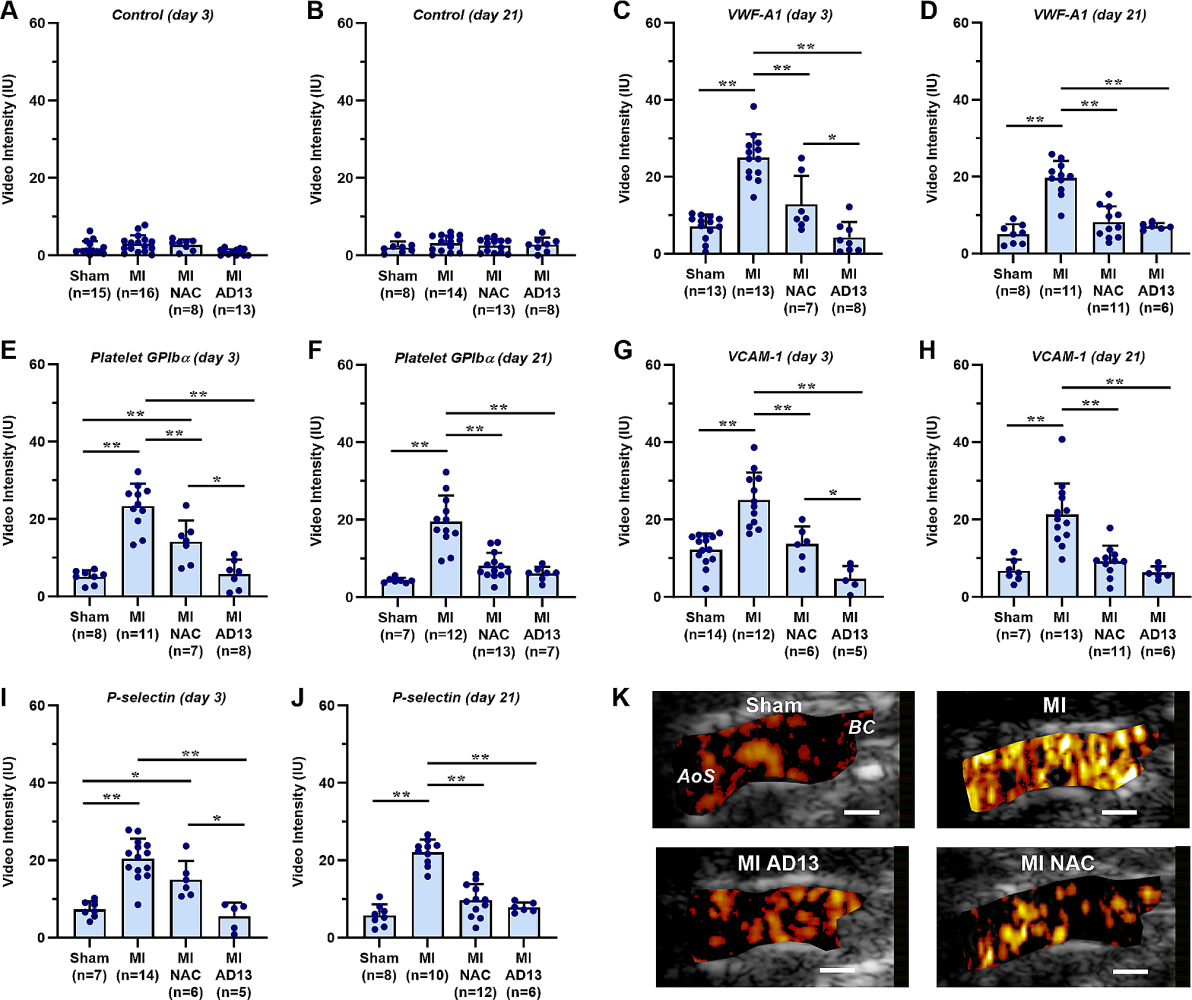
Contrast-enhanced ultrasound molecular imaging of the proximal aorta. Molecular imaging data (mean ± SEM) from day 3 and 21 are shown for animals undergoing sham surgery, MI, or MI treated with either N-acetylcysteine (*NAC*) or rADAMTS13 (*AD13*). CEUS data was performed with (**A, B**) control non-targeted microbubbles, or microbubbles targeted to: (**C, D**) VWF A-1 domain,, (**E, F**) platelet GPIbα, (**G, H**) VCAM-1, or (**I, J**) P-selectin. *p < 0.05; **p < 0.01 (by ANOVA corrected for multiple comparisons). Data shown are for mice undergoing sham suture placement, closed-chest MI, and closed-chest MI with treated with NAC or ADAMTS13. (**K**) Examples of background-subtracted color-coded CEUS molecular imaging of platelet GPIbα of the thoracic aorta (long-axis) from *LDLR*^*−/−*^*Apobec1*^*−/−*^ mice from each group at day 21. Targeted MB signal often appears in the “lumen” due to volume averaging that occurs when beam elevation dimension exceeds the aortic diameter. The location of the aortic sinuses (*AoS*) and origin of the brachiocephalic artery (*BC*) are shown in the sham-treated animal

When comparing the sham-surgery and MI groups, control microbubble signal on CEUS was uniformly low in all groups at day 3 and day 21 (Fig. 2A and B). In comparison, mice undergoing MI without VWF-targeted therapy had two- to four-fold higher molecular imaging signal for VWF, platelets, P-selectin, and VCAM-1 at day 3, which persisted until day 21 post-MI (Fig. 2C and J). Interventions targeted to reduce endothelial-associated VWF with either NAC or rADAMTS13 significantly attenuated the post-MI markers of remote plaque activation on CEUS at both day 3 and day 21 (Fig. 2C and K). At the earlier time interval (day 3), rADAMTS13 had a somewhat greater inhibitory effect than NAC, and entirely suppressed the post-MI activation response for all targets, whereas the two therapies had equal effect at day 21. Treatment with anti-FXI antibody had minimal effect on suppressing the post-MI arterial activation response on CEUS molecular imaging (Supplemental Fig. 2).

### Suppression of Post-MI Remote plaque progression by VWF-targeted interventions

**Fig. 3:**
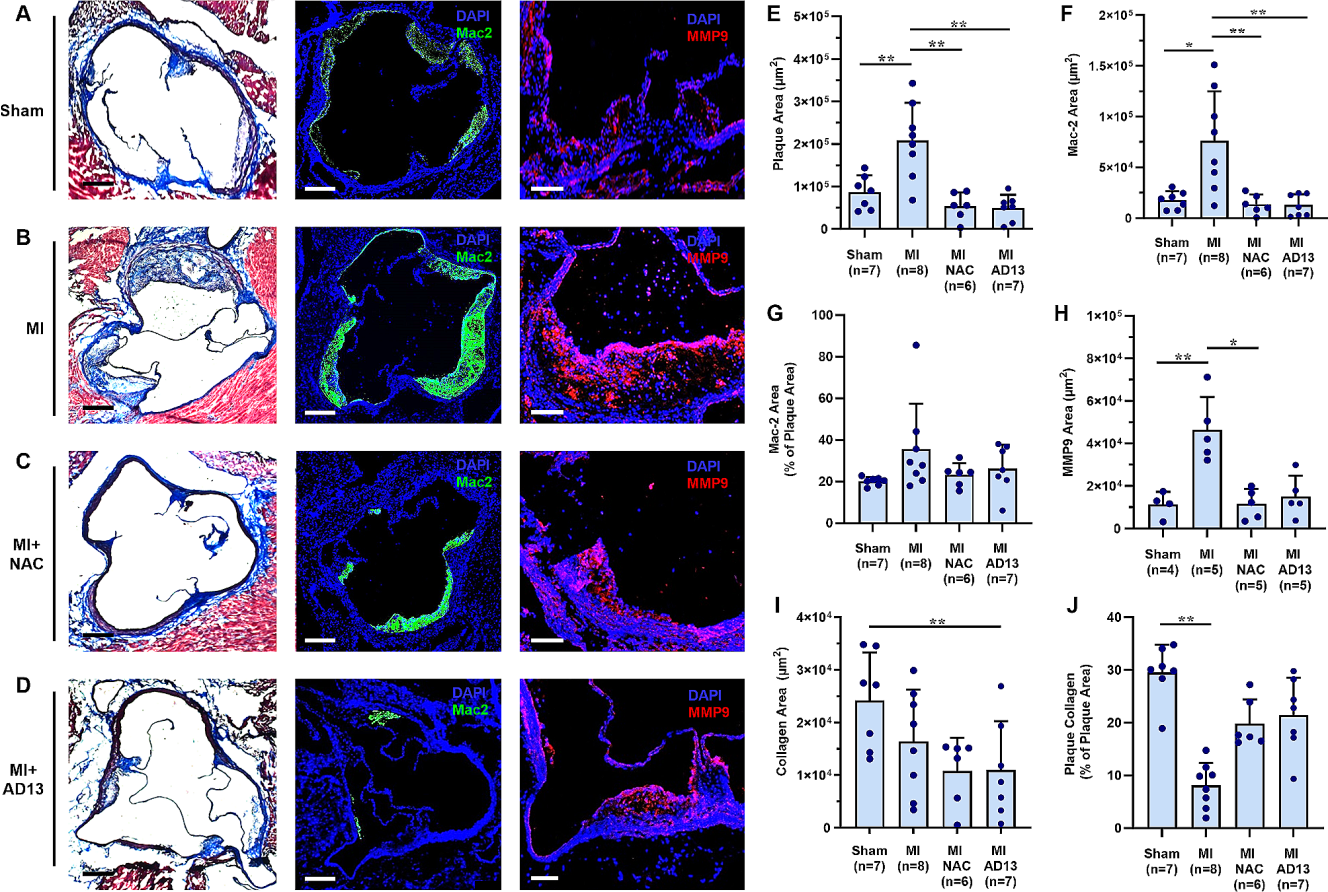
Histologic evidence for post-mi remote plaque activation. (**A to D**) Aortic root plaque histology from *LDLR*^*−/−*^*Apobec1*^*−/−*^ mice at day 21 from each of the treatment groups including Masson’s trichrome (*left panels*) for plaque size and collagen, Mac2 staining (*middle panels*), and MMP9 (*right panels*) with DAPI counterstaining (*blue*) for nuclei. Scale bars = 100 μm for Masson’s and Mac 2; and 200 μm for MMP9. Quantitative histology data (mean ± SEM) are shown for (**E**) plaque area, (**F**) plaque area positive for Mac-2, (**G**) percent of plaque area positive for Mac-2, (**H**) plaque area positive for MMP9, (**I**) plaque collagen area, and (**J**) percent of plaque area positive for collagen. *p < 0.05, **p < 0.01 (by ANOVA corrected for multiple comparisons)

At day 21, aortic root plaque size, plaque area positive for Mac2 on macrophages and phenotypically-transformed smooth muscle cells, and MMP9 protease in the necrotic core were all greater in animals undergoing MI versus sham controls, whereas the percentage of plaque area positive for Mac2 was similar (Fig. 3A and H). Treatment with either NAC or rADAMTS13 attenuated the increase in plaque size, Mac2 staining, and MMP9 after MI. Compared to sham controls, mice undergoing MI without VWF-targeted therapy had less collagen as a proportion of total plaque size only (Fig. 3I and J). Anti-FXI therapy tended to have much less of an effect on plaque size and markers of inflammation than other therapies (Supplemental Fig. 3). These data confirm that MI stimulates remote plaque rapid growth with attendant expansion of the necrotic core, all of which can be suppressed by VWF-targeted therapies.

### Evidence for pro-atherogenic platelet signaling

**Fig. 4:**
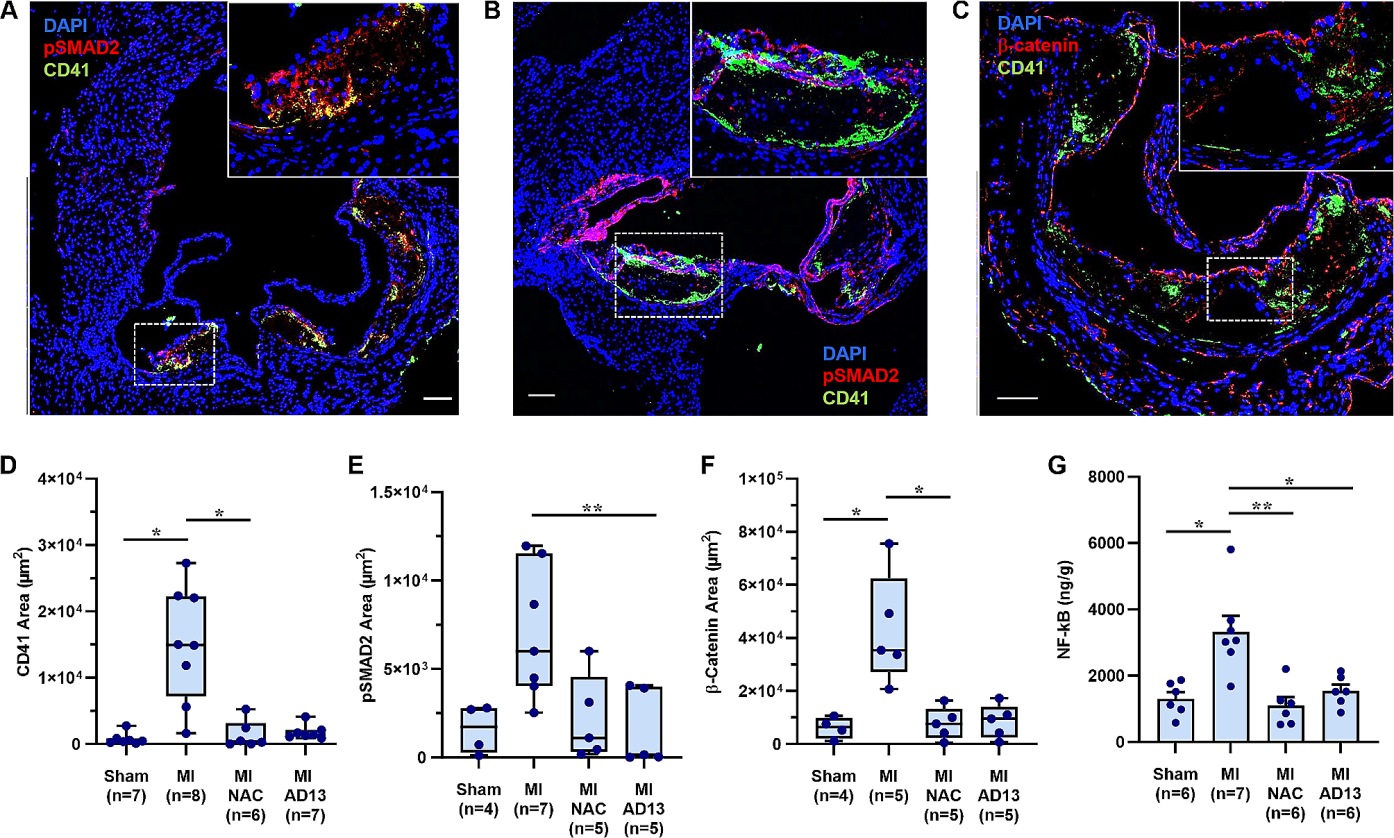
Aortic plaque platelet adhesion, platelet signaling, and inflammatory activation. (**A-C**) Examples of aortic root plaque histology for platelet CD41 and either pSMAD2 or β-catenin from three separate *LDLR*^*−/−*^*Apobec1*^*−/−*^ mice undergoing MI without VWF-targeted therapy. Insets at the upper right illustrate higher magnification of the areas within the dashed lines. Counterstaining for nuclei was performed with DAPI. Scale bar = 100 μm. Quantitative histology results (mean ± SEM) are shown for plaque area positive for: (**D**) CD41 staining, (**E**) pSMAD2, and (**F**) β-catenin. (**G**) Descending aorta NF-κB P65 component concentration by ELISA. *p < 0.05, **p < 0.01 (by Kruskal-Wallis corrected for multiple comparisons). Examples for immunofluorescent staining from other groups are provided in Supplemental Fig. 4

To corroborate CEUS molecular imaging findings, we performed aortic root histology for platelet CD41 at day 21. Compared to sham controls, mice with MI demonstrated a marked increase in platelets associated with plaque which were observed not only adhering to the endothelial surface but also in the plaque core (Fig. 4A and D, Supplemental Fig. 4). Enhanced platelet CD41 staining associated with plaques in the post-MI mice was almost entirely suppressed by NAC and rADAMTS13. Staining was performed for pSMAD2 as an activation marker of the TGFβ1 canonical signaling pathway which, despite its reparative and anti-inflammatory profile [27], can also be pro-inflammatory in atherosclerosis when endothelial-derived [28]. Staining for pSMAD2 was greater in animals undergoing MI compared with sham controls, and was suppressed in post-MI animals by NAC and rADAMTS13 (Fig. 4A and B, and 4E; Supplemental Fig. 4). In untreated post-MI mice, dual staining revealed that pSMAD2-positive cells were often co-localized with or in close association with platelets (CD41 positive). Staining was also performed for β-catenin, which influences endothelial activation status through canonical WNT signaling and can be either induced (TGFβ1) or inhibited (Dickkopf-1) by platelet-derived factors [28–30]. Staining for β-catenin was increased in animals undergoing MI and was suppressed by both NAC and rADAMTS13 (Fig. 4C and F). Staining for β-catenin was localized almost entirely to endothelial cells without convincing co-localization with CD41. Aortic NF-kB (P65 subunit) concentration at day 21 as a marker of pro-inflammatory activation was significantly higher for post-MI than sham-treated controls, and entirely suppressed post-MI by NAC and rADAMTS13 (Fig. 4G).

## Discussion

This study was designed with two main goals. The first aim was to assess whether molecular imaging of thromboinflammatory processes that occur at the blood-endothelial interface provides valuable information on the underlying processes responsible for non-culprit RPA after MI. The second goal was to apply this information to understand how therapies that suppress endothelial-associated VWF can be used to prevent remote plaque activation. Our results indicate that CEUS molecular imaging can quantify VWF-mediated platelet adhesion and subsequent endothelial pro-inflammatory activation that drive accelerated plaque growth after MI. It also provided a readout for responses on a molecular and cellular level to therapeutic interventions intended to prevent rapid plaque progression in non-culprit vessels.

Thromboinflammation and the specific role of platelet-endothelial adhesion in indolent forms of atherogenesis has been a topic of interest based on the potential to identify novel treatments. Studies, including those using CEUS molecular imaging, have consistently demonstrated the presence of excess endothelial-associated VWF and platelet adhesion at very early stages of disease [12, 31, 32]. In murine models of hyperlipidemia, the subsequent development of atherosclerotic lesions can be slowed either by inhibiting GPIbα, the platelet ligand for VWF, or by genetic deletion of VWF [31, 33]. Moreover, atherosclerosis is accelerated and adhesion molecule expression is increased with genetic deletion of ADAMTS13 which cleaves VWF at the A2 domain and removes it from the endothelial surface [3, 34]. Combined, these findings suggest a role for VWF-mediated platelet adhesion and paracrine signaling in atherogenesis.

Because molecular imaging provides a non-invasive temporal assessment of plaque phenotype, CEUS molecular imaging has been used to also study remote plaque activation after MI. These studies were the first to establish that accelerated growth of non-culprit plaques after MI was associated with a sudden increase in not only endothelial adhesion molecular expression, but also endothelial-associated VWF and platelet adhesion [9]. These markers of remote plaque activation were sustained in mice with hyperlipidemia, which can be explained by recent data that indicate that LDL cholesterol enhances the self-association and therefore adhesive capacity of VWF [35].

In the current study, using a closed-chest model of MI and CEUS molecular imaging in hyperlipidemic mice, we again demonstrated sustained endothelial VWF, platelet adhesion, and inflammatory activation at a remote plaque site which was associated with a 2- to 3-fold increase in plaque size and macrophage content when assessed 3 weeks after MI. Both rADAMTS13 and NAC not only suppressed the post-MI increase in plaque VWF and platelet adhesion, but also endothelial P-selectin and VCAM-1. VWF-targeted therapies also prevented the post-MI accelerated plaque growth, inflammation, protease activity, and NF-κB activation. These data implicate VWF-mediated platelet adhesion as a key event for stimulating adverse remote plaque morphologic changes in non-culprit vessels after MI. On molecular imaging, rADAMTS13 had a greater effect than NAC at reducing plaque thromboinflammation only at the early time point (day 3), likely owing to its parenteral administration and greater early bioavailability. This advantage of rADAMTS13 was not sustained and the two therapies were equally effective at preventing remote plaque growth. While inhibition of FXI did not show beneficial effect, the data from this group are useful for linking plaque phenotypic response on CEUS molecular imaging to morphologic outcomes. The molecular imaging studies also strongly suggest that the appearance of endothelial-surface VWF is a proximal event in the rapid progression of remote plaques because its removal from the endothelial surface also prevented later expression of VCAM-1 and P-selectin and other manifestations of plaque progression. Prior studies have indicated that these events are not reflected by circulating levels of VWF [36], thereby requiring molecular imaging of events on the endothelial cell surface.

Plaque histologic staining was performed for β-catenin and pSMAD2. These studies were not intended to implicate these pathways in atherogenesis, which is controversial. Rather they were simply used as evidence for platelet signaling through TGFβ1 and were elevated post-MI and suppressed by therapies that were effective at reducing endothelial VWF and thromboinflammation. An interesting finding that has been noted previously was that platelet staining was observed not only on the plaque surface, but also in the plaque interior, suggesting a mechanism for internalization of platelet material since this murine model is not characterized by plaque hemorrhage.

### Study limitations

There are limitations of the study to highlight. With respect to mechanism, the platelet-derived pathways responsible for plaque activation were not investigated. Also, reasons why benefit was seen with administration of exogenous rADAMTS13 beyond that which naturally occurs in mice is not entirely clear. However, studies have indicated that high oxidative stress, which occurs in hypercholesterolemia and MI, can impair ADAMTS13 activity and removal of VWF from the endothelial surface [9, 13, 37], providing an explanation for the effects of additional enzyme. We selected a single time point to evaluate accelerated plaque growth, although previous studies have indicated that remote plaque activation continues for up to 3 months [9]. The study was not performed in the context of standard secondary prevention therapies routinely given after MI. Yet recent data using serial invasive assessment of non-culprit coronary plaques in patients indicates that standard therapy, including intensive lipid lowering, has very modest effects at improving plaque volume or lipid core burden when used after recent MI [38], indicating a possible role for new approaches. Finally use of murine models, and in this case small numbers of animals, to represent a human condition is always a limitation.

### Conclusions

In summary, CEUS molecular imaging in this pre-clinical model of MI was able to characterize novel and modifiable thromboinflammatory processes that are responsible for remote plaque activation after a focal ischemic event. Multi-target imaging in conjunction with therapies to enhance removal of endothelial surface-associated VWF revealed that plaque inflammatory activation is strongly influenced by VWF-mediated platelet adhesion to the endothelium. These findings support investigation into new secondary therapies for reducing non-culprit artery events after MI, and the use of molecular imaging in hypothesis testing for new therapies in atherosclerotic disease.

### Electronic supplementary material

Below is the link to the electronic supplementary material.

Supplementary Material 1

Supplementary Material 2

Supplementary Material 3

Supplementary Material 4

Supplementary Material 5

Supplementary Material 6

Supplementary Material 7

## Data Availability

The datasets used and/or analyzed during the current study are available from the corresponding author on reasonable request.

## Abbreviations

ADAMTS13: A Disintegrin-like and Metalloprotease domain with Thrombospondin type I motifs
CEUS: Contrast-enhanced ultrasound
FXI: Factor XI
GCS: Global circumferential strain
GLS: Global longitudinal strain
GPIbα: Glycoprotein-Ibα
LAD: Left anterior descending coronary artery
LVEF: Left ventricular ejection fraction
MMP: Matrix metalloproteinase
NAC: N-acetylcysteine
pSMAD2: Phosphorylated SMAD2
TGFβ1: Transforming growth factor-β1
VCAM-1: Vascular cell adhesion molecule-1
VWF: von Willebrand factor
