# Supplementary material for: Removal of endothelial surface-associated von villebrand factor suppresses accelerate datherosclerosis after myocardial infarction
Source: J Transl Med. 2024 May 1;22:412. doi: 10.1186/s12967-024-05231-6 (PMC11062912; doi:10.1186/s12967-024-05231-6)
Supplement: Supplementary file 2 — Supplementary Material 2 [file 12967_2024_5231_MOESM2_ESM.pdf]

SUPPLEMENTAL FIGURES

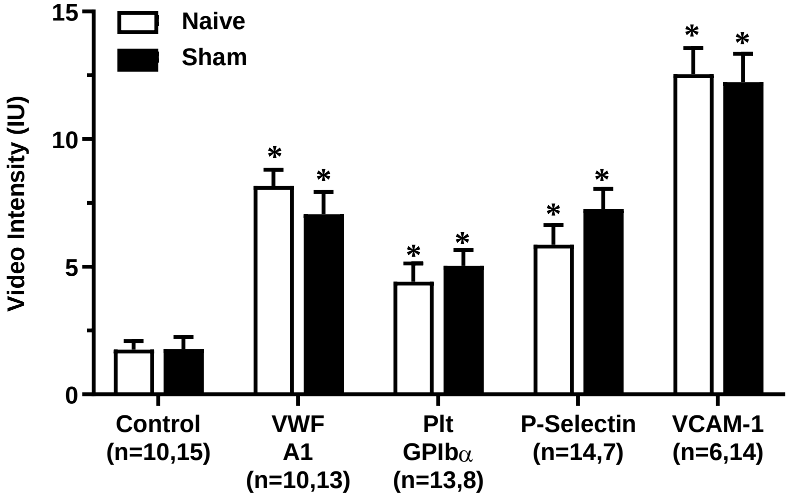

Supplemental Figure 1. CEUS Molecular Imaging From All Control Groups. Molecular imaging results are shown for control microbubbles and each targeted agent from naïve mice not undergoing suture placement, and sham-treated *LDLR*$^{-/-}$*Apobec1*$^{-/-}$ mice undergoing suture but without ischemia. The number of mice (n) in the naïve and sham-treated groups, respectively, are shown for each targeted agent. *p<0.05 vs control microbubbles within the same group (corrected for multiple comparisons).

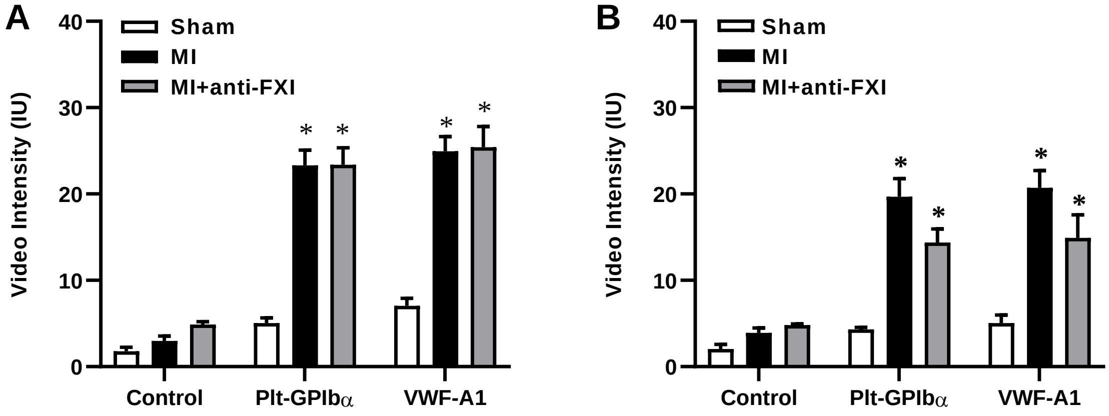

Supplemental Figure 2. Effect of FXI Inhibition on Endothelial Phenotype by CEUS Molecular Imaging. CEUS data are shown for platelet GPIbα and VWF A1 domain at (**A**) day 3, and (**B**) day 21 inclusive of data from post-MI *LDLR*$^{-/-}$*Apobec1*$^{-/-}$ mice treated with anti-FXI. *$p<0.05$ versus sham and versus control agent.

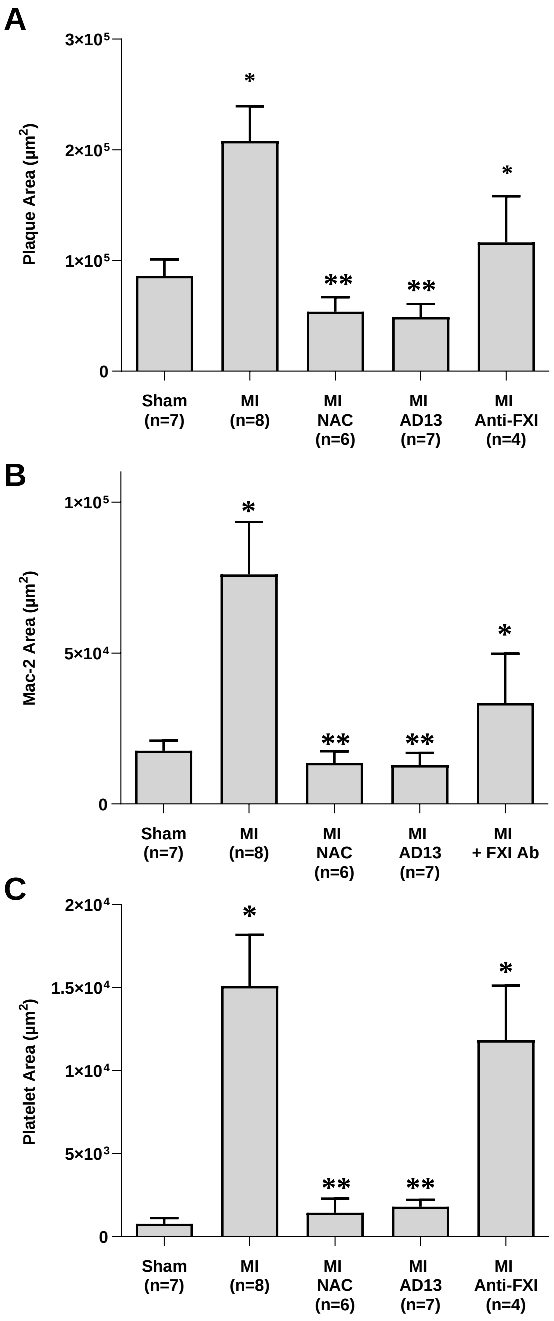

Supplemental Figure 3. Quantitative Aortic Root Histology Inclusive of FXI inhibition Data Histology results inclusive of data from post-MI *LDLR*$^{-/-}$*Apobec1*$^{-/-}$ mice treated with anti-FXI for (**A**) plaque area, (**B**) total plaque area positive for Mac-2, and area staining positive for platelet CD41.*p<0.05 vs sham; **p<0.05 vs MI.

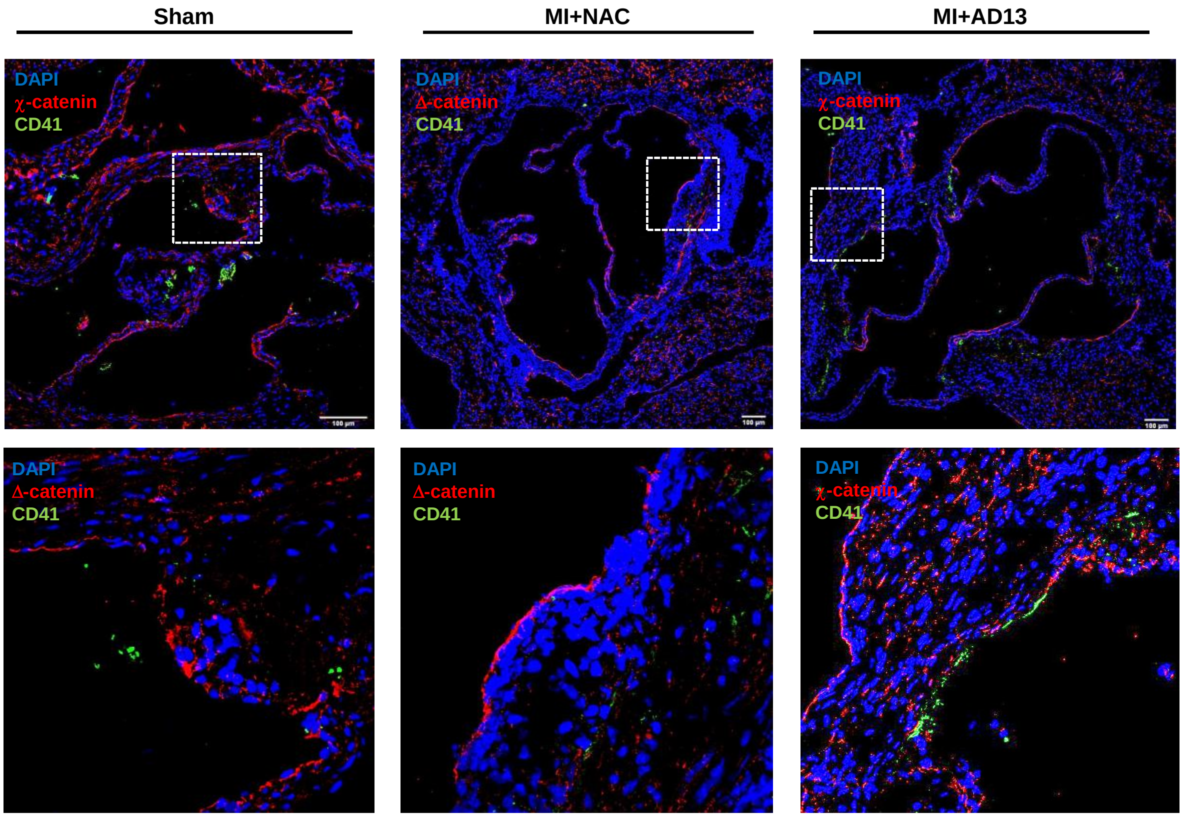

Supplemental Figure 4. Examples of immunofluorescence for β-catenin and CD41 from *LDLR$^{-/-}$Apobec1$^{-/-}$* mice undergoing sham procedure, or undergoing MI with therapies targeted to VWF. DAPI counterstaining was performed for nuclei. Images at the bottom represent higher magnification for the regions defined by the dashed lines. Scale bars = 100 µm. Examples from animals undergoing MI without VWF-targeted therapy are shown in Figure 4.
