# Supplementary material for: Removal of endothelial surface-associated von villebrand factor suppresses accelerate datherosclerosis after myocardial infarction
Source: J Transl Med. 2024 May 1;22:412. doi: 10.1186/s12967-024-05231-6 (PMC11062912; doi:10.1186/s12967-024-05231-6)
Supplement: Supplementary file 7 — Supplementary Material 7 [file 12967_2024_5231_MOESM7_ESM.docx]

**VIDEO LEGENDS**

**Video 1** Example of two-dimensional echocardiography in the parasternal long-axis view from a mouse at baseline prior to LAD ischemia.

**Video 2** Example of two-dimensional echocardiography in the mid-ventricular parasternal short-axis view at baseline prior to LAD ischemia from the same mouse as Video 1.

**Video 3** Example of two-dimensional echocardiography in the parasternal long-axis view during LAD ischemia from the same mouse in Video 1 and 2.

**Video 3** Example of two-dimensional echocardiography in the parasternal short-axis view during LAD ischemia from the same mouse in Video 1 and 2.
