# Supplementary material for: Removal of endothelial surface-associated von villebrand factor suppresses accelerate datherosclerosis after myocardial infarction
Source: J Transl Med. 2024 May 1;22:412. doi: 10.1186/s12967-024-05231-6 (PMC11062912; doi:10.1186/s12967-024-05231-6)
Supplement: Supplementary file 1 — Supplementary Material 1 [file 12967_2024_5231_MOESM1_ESM.docx]

**Jonathan R. Lindner, M.D.**

*Frances Myers Ball Chair of Medicine*

*Vice Chief for Research, Cardiovascular Division*

University of Virginia Medical Center

Robert M. Berne Cardiovascular Research Center – MR5

415 Lane Rd, Box 801394

Charlottesville, VA 22903

Tel. (434) 297-9442

January 30, 2024

Francesco Marincola, MD

Editor-in-Chief, *Journal of Translational Medicine*

**RE: MS Submission**

Dear Dr. Marincola

On behalf of all authors, I am pleased to submit this second revision of a manuscript for consideration for publication in *Journal of Translational Medicine.* We believe this study to be extremely impactful for several reasons. First, we have used endothelial molecular imaging of thromboinflammation, which is uniquely able to characterize events in vivo on the endothelial surface, to investigate novel modifiable mechanisms for remote non-culprit plaque activation after MI. By using multi-target molecular imaging, we were able to show the critical importance of von Willebrand factor (VWF) -mediated platelet adhesion in triggering remote plaque activation and inflammation. Additionally, we showed for the first time that therapies that are effective at removing VWF from the endothelial surface almost entirely suppress remote plaque inflammatory activation and accelerated growth after MI. I believe these results will be highly cited in the future by specialists in atherosclerosis biology based on the extent of effect downstream from endothelial surface-associated VWF. We have selected *Journal of Translational Medicine* based on the direct clinical impact of these findings, which have led us to initiate NIH funding for a clinical trial aimed at suppressing these processes.

We confirm that: 1) The paper is not under consideration elsewhere; 2) none of the paper’s contents, with the exception of abstracts, have been previously published; 3) all authors have read and approved the manuscript. There are no disclosures with respect to relations to industry or other conflicts of interest (as stated). All authors have given final approval of the manuscript. All authors were involved in data generation, data interpretation/analysis, and manuscript preparation. Dr.’s Lindner and Lopez were responsible for study design.

We hope that the reviewers and editors will consider this revised manuscript worthy of publication in the *Journal of Translational Medicine* and look forward to hearing from you soon.

Warm regards,

Jonathan R. Lindner, MD, FACC, FASE
